# Overexpression of LINC00160 predicts poor outcome and promotes progression of clear cell renal cell carcinoma

**DOI:** 10.18632/aging.103091

**Published:** 2020-04-21

**Authors:** Gong Cheng, Yi Yu, Longwang Wang, Qiufeng Pan

**Affiliations:** 1 Department of Urology, Union Hospital, Tongji Medical College, Huazhong University of Science and Technology, Wuhan 430022, China; 2 Department of Urology, The Second Affiliated Hospital of Nanchang University, Nanchang 330006, China; 3 Department of Urology, The First Affiliated Hospital of Nanchang University, Nanchang 330006, China

**Keywords:** LINC00160, renal cancer, biomarker, prognosis

## Abstract

Clear cell renal cell carcinoma (ccRCC) is the most common subtype of renal carcinoma and exhibits a high risk of invasion and metastasis. It is urgent to uncover a novel biomarker and clarify the underlying mechanism for ccRCC progression and metastasis. Although accumulating research has demonstrated that long non-coding RNAs (lncRNAs) play crucial roles in tumor progression, numerous lncRNAs in ccRCC are largely unknown. Therefore, we screened the differentially expressed lncRNAs among several GEO datasets and chose LNC00160 for further investigation. LNC00160 was significantly upregulated in ccRCC and high expression predicted poor prognosis; higher expression of LNC00160 was associated with advanced clinic pathological parameters in TCGA_KIRC Cohort. Knockdown of LNC00160 suppressed malignancy of ccRCC in *vitro* and in *vivo*. Correlation analysis and gene set enrichment analysis (GSEA) revealed that LNC00160 might be associated with Wnt signaling pathway, mTOR signaling pathway, fatty acid metabolism and cell cycle. In conclusion, our results demonstrated that LNC00160 acted as an oncogenic gene and a specific prognostic indicator for patients with ccRCC, and that LNC00160 might be a targeted intervention for ccRCC patients in the future.

## INTRODUCTION

Cancer is the second leading cause of deaths after heart disease regardless of patient sex in the United States; in 2019, there are 73820 estimated new cases and 14770 estimated deaths from kidney malignancy [1]. Clear cell renal cell carcinoma (ccRCC), the most common malignancy in kidney, exhibits a high heterogeneity and risk of metastasis and management of which is based on tumor progression [2]. Standard surgical radical nephrectomy or partial nephrectomy are the most optimal treatment for localized ccRCC; however, a portion of patient already have distant metastasis when diagnosed and thus lose the chance of radical surgery; these patients with metastasis have no alternative but to turn to tyrosine kinase inhibitor (TKI) based chemotherapy [3–5]. Unfortunately, because of the subsequent acquired drug resistance, TKI-treated patients still develop poor clinical outcome [6]. Timely diagnosis and treatment will be vital for the improvement of patients’ survival [7]. Therefore, exploring novel effective prognostic biomarker and their underlying mechanism in tumor progression and drug resistance will lead to a better understanding of tumor biology and pave the way for the treatment of ccRCC [8].

The long noncoding RNAs (lncRNAs) are defined and classified according to their non-protein-coding characteristic and the arbitrary cut-off of 200 nucleotides [9, 10]. Accumulating and compelling evidence in the past decade indicated that lncRNAs are always altered in human urologic tumors and play important roles in cancer progression and metastasis; moreover, they participate in the process of chemotherapy resistance [11, 12]. Although numerous lncRNAs have been unearthed and investigated, many facets of their biology in ccRCC remain largely unknown [13].

In the present study, through screening the differentially expressed lncRNAs across several gene sets from the GEO database, we finally selected LINC00160 for subsequent investigation, including its prognostic role and correlation with clinicopathological characteristics; furthermore, we performed experimental research in *vitro* and *vivo* to elucidate the functional effect on ccRCC.

## RESULTS

### LINC00160 exhibits a diagnostic and prognostic value in renal cell carcinoma

**Figure 1:**
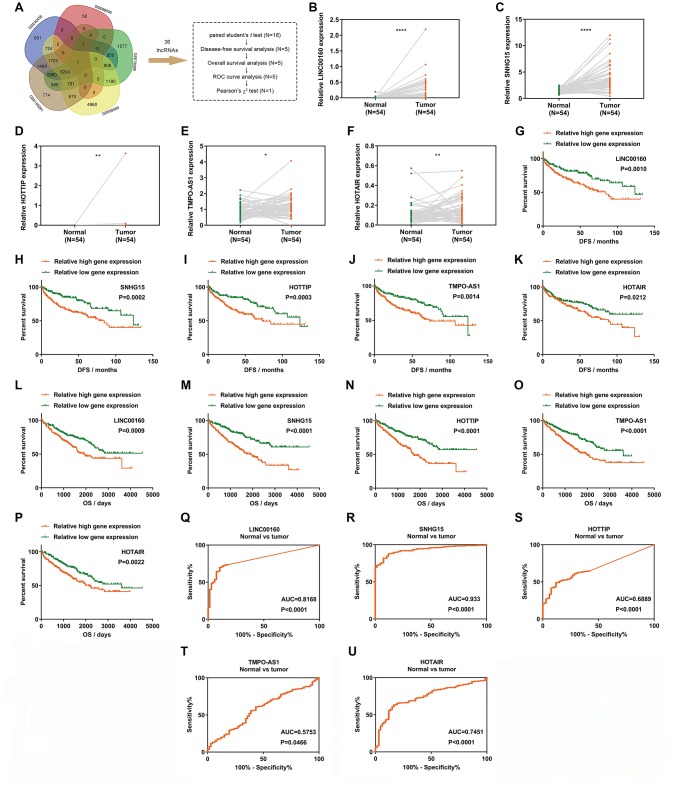
**LINC00160 exhibits a diagnostic and prognostic value in renal cell carcinoma.** (**A**) A Venn diagram of 5 independent gene sets (differentially expressed genes in RCC) from GEO datasets. (**B**–**F**) Relative gene expression comparison of LINC00160 (**B**), SNHG15 (**C**), HOTTIP (**D**), TMPO-AS1 (**E**) and HOTAIR (**F**) from 54 paired tissues in the TCGA-KIRC database. (**G**–**K**) DFS of RCC patients was associated with LINC00160 (**G**), SNHG15 (**H**), HOTTIP (**I**), TMPO-AS1 (**J**) and HOTAIR (**K**) expression. (**L**–**P**) OS of RCC patients was associated with LINC00160 (**L**), SNHG15 (**M**), HOTTIP (**N**), TMPO-AS1 (**O**) and HOTAIR (**P**) expression. (**Q**–**U**) ROC analysis was performed to differentiate kidney cancer patients from healthy individuals, according to LINC00160 (**Q**), SNHG15 (**R**), HOTTIP (**S**), TMPO-AS1 (**T**) and HOTAIR (**U**) expression. *P<0.05, **P<0.01, ***P<0.001 and ****P<0.0001. DFS, disease-free survival rate; OS, overall survival rate; ROC, receiver operating characteristic curve; RCC, renal cell carcinoma.

Three independent cancer progression-related gene sets (namely GSE40435, GSE89563 and GSE105261) and two independent sunitinib resistance-related gene sets (namely GSE69535 and GSE76068) (Figure 1A) were used for screening after GEO2R analysis [14–18]. Thirty-six upregulated candidate lncRNAs were retrieved from four to five GSE profiles, which indicated that these differentially expressed genes could distinguish ccRCC from normal tissues and discriminate sunitinib resistant cells from parental cells. Fifty-four paired normal and tumor samples were examined using paired student's *t*-tests to validate lncRNA relative expression levels. Out of the 36 upregulated lncRNAs, a statistically significant difference was identified in 16 lncRNAs (Figure 1B–1F, Supplementary Figure 1). To determine which lncRNAs were associated with the prognosis of cancer patients, 16 upregulated lncRNAs were analyzed in relation to disease-free survival (DFS). Out of 16 upregulated lncRNAs, 5 exhibited statistical difference between relative high gene expression and low expression groups in DFS (Figure 1G–1K). All 5 upregulated lncRNAs passed the statistical tests in overall survival (OS) analysis (Figure 1L–1P). To further investigate the diagnostic value of 5 candidate lncRNAs in ccRCC, receiver operating characteristic curve (ROC) analysis was performed to differentiate kidney cancer patients from healthy individuals, according to lncRNA expression. Out of 5 genes, all exhibited statistically diagnostic values (P<0.05; Figure 1Q–1U). Considering known effects of SNHG15, HOTTIP and HOTAIR in ccRCC [19–21], we focused our attention on LINC00160 (areas under the curve, AUC=0.8168) rather than TMPO-AS1 (AUC=0.5753). To obtain more detailed information about LINC00160, ROC curves were used to distinguish between the G1+G2 and G3+G4, M0 and M1, N0 and N1, T1+T2 and T3+T4, and Stage I+II and Stage III+IV patient samples (Supplementary Figure 2A–2E).

### Correlation of LINC00160 expression and overall and disease-free survival in subgroup patients of ccRCC

**Figure 2:**
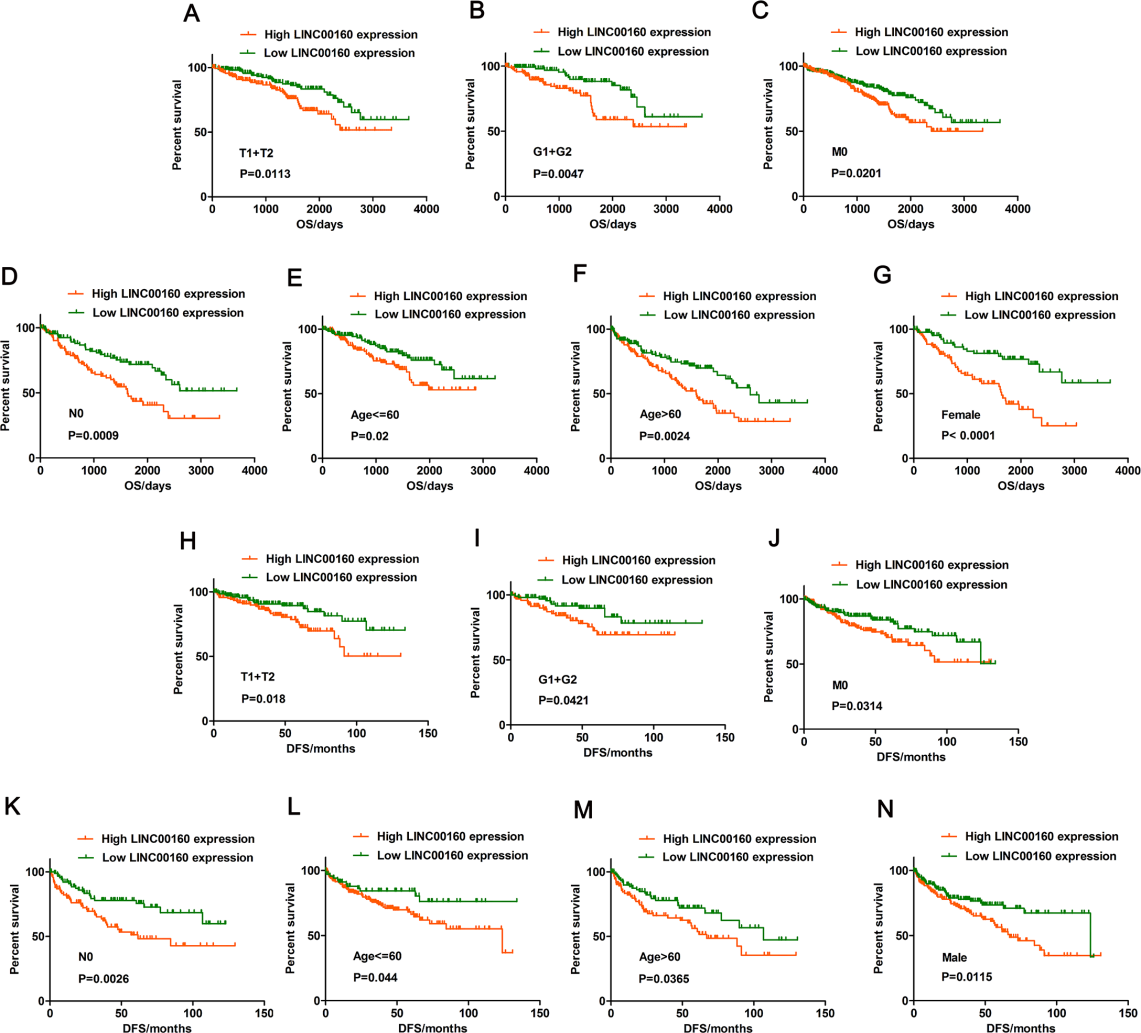
**Prognostic significance of LINC00160 in TCGA-KIRC datasets.** (**A**–**G**) Overall survival analysis towards the expression of LINC00160 was performed in subgroups of patients with ccRCC: (**A**) T1+T2 stage, (**B**) G1+G2 stage, (**C**) M0 stage, (**D**) N0 stage, (**E**) Age <=60, (**F**) Age >60, (**G**) Female; (**H**–**N**) Disease-free survival analysis towards the expression of LINC00160 was performed in subgroups of patients with ccRCC: (**H**) T1+T2 stage, (**I**) G1+G2 stage, (**J**) M0 stage, (**K**) N0 stage, (**L**) Age <=60, (**M**) Age >60, (**N**) Female. TCGA-KIRC, The Cancer Genome Atlas Kidney renal clear cell carcinoma; ccRCC, clear cell renal cell carcinoma; OS, overall survival; DFS, disease-free survival; *P < 0.05, ** *P* < 0.01, ***P < 0.001, and ****P < 0.0001.

Kaplan-Meier survival analysis was performed to investigate the correlation of LINC00160 expression and OS and DFS in subgroup patients with ccRCC. various subgroups OS and DFS analysis demonstrated that LINC00160 expression could acted as a potential prognostic factor for patients including T1+T2 stage (Figure 2A, 2H), G1+G2 stage (Figure 2B, 2I), M0 (Figure 2C, 2J), N0 (Figure 2D, 2K), Age <=60 (Figure 2E, 2L), Age >60 (Figure 2F, 2M), Female (Figure 2G), and Male (Figure 2N).

### LINC00160 was associated with various types of clinicopathological parameters in ccRCC tissues

**Figure 3:**
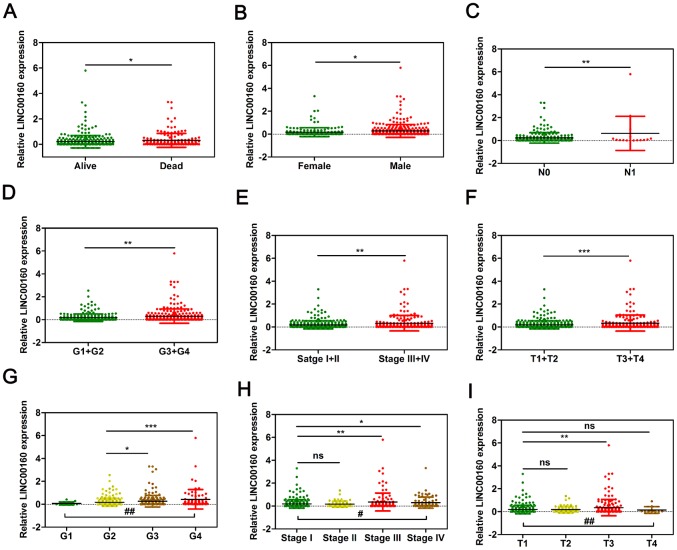
**LINC00160 expression was closely associated with various clinicopathological parameters in TCGA-KIRC microarray datasets.** The mRNA levels of LINC00160 were compared in different clinicopathological characteristics: (**A**) Dead versus Alive, (**B**) Female versus Male, (**C**) N stage, (**D**, **G**) Fuhrman grade, (**E**, **H**) stage, (**F**, **I**) T. TCGA-KIRC, The Cancer Genome Atlas Kidney renal clear cell carcinoma. *P < 0.05, ** *P* < 0.01, ***P < 0.001, and ****P < 0.0001, # *P* < 0.05, ## *P* < 0.01. #, one-way ANOVA; *, *t* test.

To determine whether LINC00160 were closely associated with clinicopathological factors of kidney cancer patients, Pearson’s χ^2^ test was performed with age, gender, T, N, M, grade and stage (Table 1). It was shown that LINC00160 was correlated with gender (P=0.0018), T (P=0.0056), M (P=0.0062), grade (P=0.0066) and stage (P=0.0018). To visualize and clarify these relationships, we divided patient samples into two subgroups (alive vs dead, female vs male, T1+T2 vs T3+T4, N0 vs N1, G1+G2 vs G3+G4, Stage I+II vs Stage III+IV). As shown in Figure 3, male and dead patients and advanced lymph node, T, grade and stage patients exhibited higher LINC00160 expression levels.

**Table 1:** The characteristics of LINC00160 in renal cell carcinoma.

**Characteristic**		**lncRNA LINC00160**		**P-value**
		**Low(n=223)**	**High(n=222)**	
**Age (years)**	<=60	103	116	0.2008
	>60	120	106	
**Gender**	Female	96	64	***0.0018***
	Male	127	158	
**T**	T1+T2	152	123	***0.0056***
	T3+T4	71	99	
**N**	N0	216	215	0.9932
	N1	7	7	
**M**	M0	198	176	***0.0062***
	M1	25	46	
**Grade**	G1+G2	116	87	***0.0066***
	G3+G4	107	135	
**Stage**	S1+S2	146	113	***0.0018***
	S3+S4	77	109	

### LINC00160 exhibits non-coding potency in RCC

**Figure 4:**
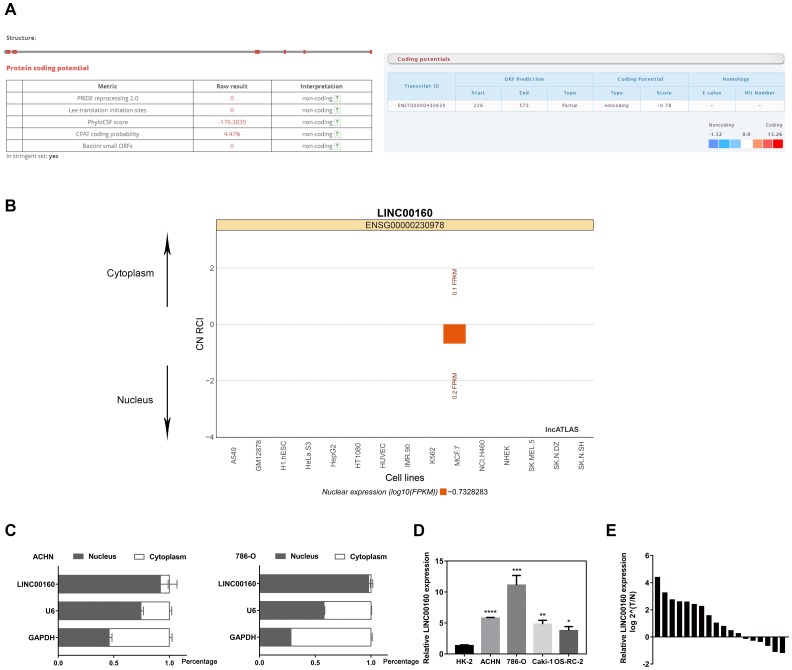
Characteristics of LINC00160 (**A**) LNCipedia and lncRNAtor websites found LINC00160 with no protein coding ability. (**B**) lncATLAS website revealed LINC00160 was mainly located in nucleus. (**C**) Separation of nuclear and cytoplasmic RNA assays indicated that LINC00160 was predominantly localized in nucleus rather than cytoplasm. (**D**) LINC00160 mRNA expression was significantly higher in RCC cell lines (ACHN, 786-O, Caki-1, OS-RC-2) compared with the kidney control cell line (HK-2). (**E**) LINC00160 mRNA level was statistically upregulated in tumor samples than adjacent normal tissues.

To assure non-coding potential of LINC00160, we screened on LNCipedia and lncRNAtor websites and found LINC00160 with no protein coding ability (Figure 4A). LncRNAs exhibit various modulatory roles through different mechanisms, largely depending on subcellular localization [22, 23]. Prediction on lncATLAS website (Figure 4B) revealed that LINC00160 was predominantly localized in nucleus rather than cytoplasm, which was further verified by our separation of nuclear and cytoplasmic RNA assays (Figure 4C), indicating that LINC00160 might behave in a cis-/trans- acting manner according to its endogenous loci with other genes. As shown in Figure 1A, 1B, we have illustrated that LINC00160 was upregulated in kidney cancer samples. To verify these results from public databases, LINC00160 mRNA expression was examined in RCC cell lines (ACHN, 786-O, Caki-1, OS-RC-2) with significantly higher levels compared to the kidney control cell line (HK-2) (Figure 4D). Moreover, patient tumor tissues were collected from Department of Urology, Union Hospital (Wuhan, China) and LINC00160 mRNA level was statistically upregulated in tumor samples than adjacent normal tissues (Figure 4E).

### LINC00160 promotes proliferation, migration and invasion of RCC

**Figure 5:**
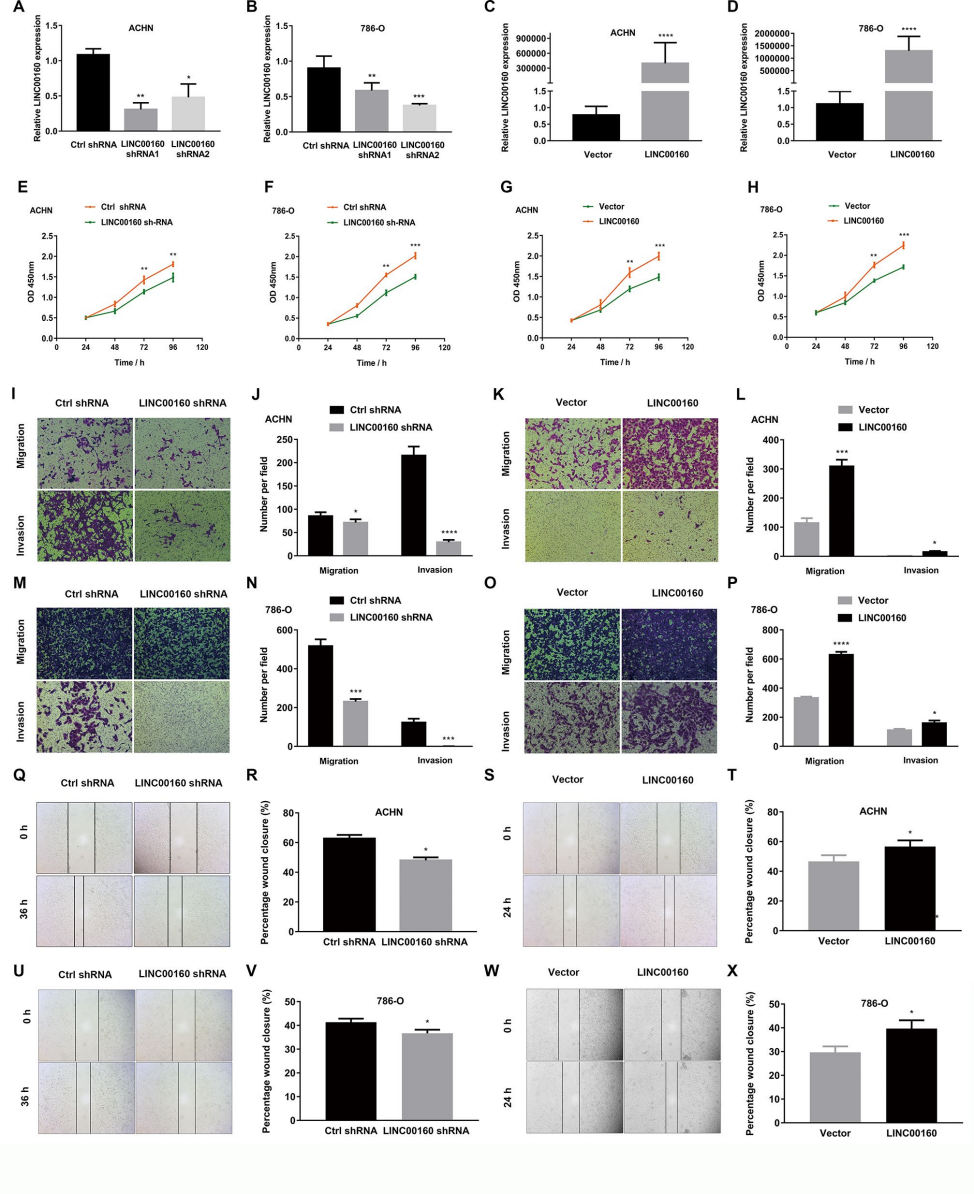
**LINC00160 promotes proliferation, migration and invasion of RCC.** (**A**–**D**) LINC00160 expression was verified after transfection in ACHN and 786-O. (**E**–**H**) Proliferation assays revealed attenuated capabilities of ACHN (**E**) and 786-O (**F**) after knocking down LINC00160, and enhanced capabilities of ACHN (**G**) and 786-O (**H**) after overexpressing LINC00160. (**I**–**L**) Migration and invasion abilities of ACHN were weakened after knocking down LINC00160, and elevated after overexpressing LINC00160. (**M**–**P**) Migration and invasion abilities of 786-O were weakened after knocking down LINC00160, and elevated after overexpressing LINC00160. (**Q**–**T**) Wound healing assays revealed impaired capabilities of ACHN after knocking down LINC00160, and promoted capabilities after overexpressing LINC00160. (**U**–**X**) Wound healing assays revealed impaired capabilities of 786-O after knocking down LINC00160, and enhanced capabilities after overexpressing LINC00160. Each experiment was performed at least three times and data was represented as mean ± SEM. *P<0.05, **P<0.01, ***P<0.001 and ****P<0.0001. RCC, renal cell carcinoma.

LINC00160 dysregulation in RCC suggested that LINC00160 might influence the progression of RCC. To test this hypothesis, we successfully constructed ACHN and 786-O cell lines with knocked down LINC00160 by transfecting short hairpin RNA (shRNA) and also constructed ACHN and 786-O with overexpressed LINC00160 by transfecting plasmid (Figure 5A–5D). CCK-8 assays were used to assess proliferation abilities of RCC cells. As shown in Figure 5E, 5F, proliferation rate of ACHN and 786-O cells was significantly repressed when knocking down LINC00160 levels. Conversely, proliferation rates have increased in LINC00160 overexpressing cells (Figure 5G, 5H). Transwell and wound healing assays were performed to assess cell migration and invasion abilities. It was obvious that migration and invasion capabilities were suppressed in LINC00160 knocked down cells (Figure 5I, 5J, 5M, 5N, 5Q, 5R, 5U, 5V), while cells with overexpressed LINC00160 showed enhanced migration and invasion abilities (Figure 5K, 5L, 5O, 5P, 5S, 5T, 5W, 5X).

### Correlation analysis between LINC00160 and protein-coding genes in TCGA-KIRC cohort and biological pathogenesis of LINC00160 in ccRCC

**Figure 6:**
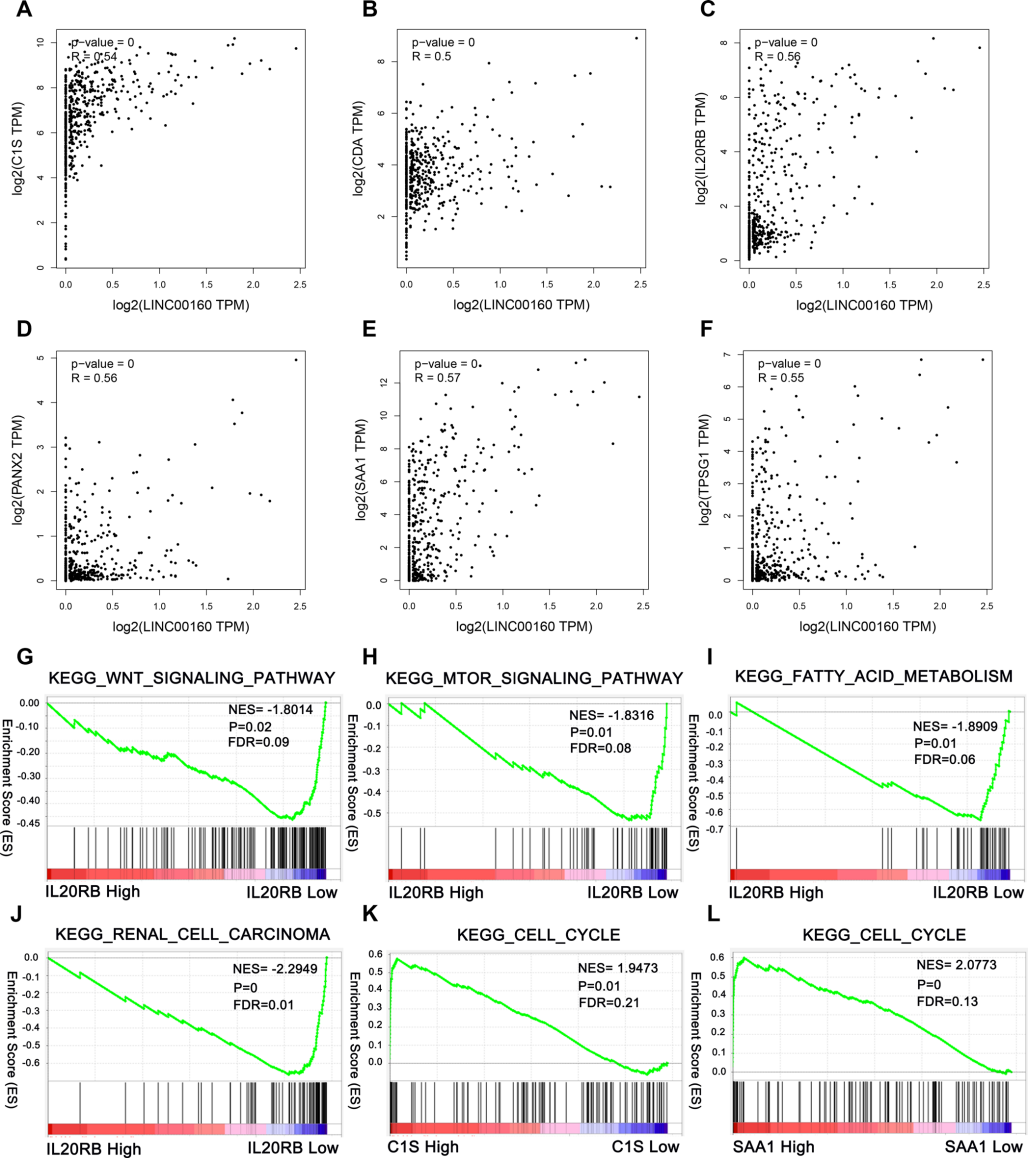
**Correlation analysis between LINC00160 and protein-coding genes in TCGA-KIRC cohort and pathways involved in the pathogenesis of LINC00160 in TCGA-KIRC with GSEA.** Correlation plots are shown with correlation coefficient more than 0.5. (**A**) C1S, (**B**) CDA, (**C**) IL20RB, (**D**) PANX2, (**E**) SAA1, (**F**) TPSG1. Enrichment curves are shown for activated gene sets related to (**G**) Wnt signaling pathway, (**H**) mTOR signaling pathway, (**I**) Fatty acid metabolism, (**J**) Renal cell carcinoma, (**K**) Cell cycle, (**L**) Cell cycle. TCGA-KIRC, The Cancer Genome Atlas Kidney renal clear cell carcinoma. FDR, false discovery rate. GSEA, gene set enrichment analysis.

We conducted the correlation analysis between LINC00160 and protein-coding genes and retrieved correlation plot with correlation coefficient higher than 0.5 in the GEPIA database (http://gepia.cancer-pku.cn/), including C1S, CDA, IL20RB, PANX2, SAA1, and TPSG1 (Figure 6A–6F). As LINC00160 was upregulated and a prognostic factor for OS and DFS, we investigated how LINC00160 was involved in ccRCC pathogenesis. Therefore, we preformed analysis of LINC00160 related protein based on GSEA, which is a computational tool to acquire statistically significant of the biological pathway to a gene set. The results revealed that low expression of IL20RB was closely associated with Wnt, mTOR signaling pathway, fatty acid metabolism, and renal cell carcinoma (Figure 6G–6J). High expression of C1S and SAA1 were enriched in cell cycle (Figure 6K, 6L).

### The knockdown of LINC00160 levels inhibits tumorigenesis in *vivo*

**Figure 7:**
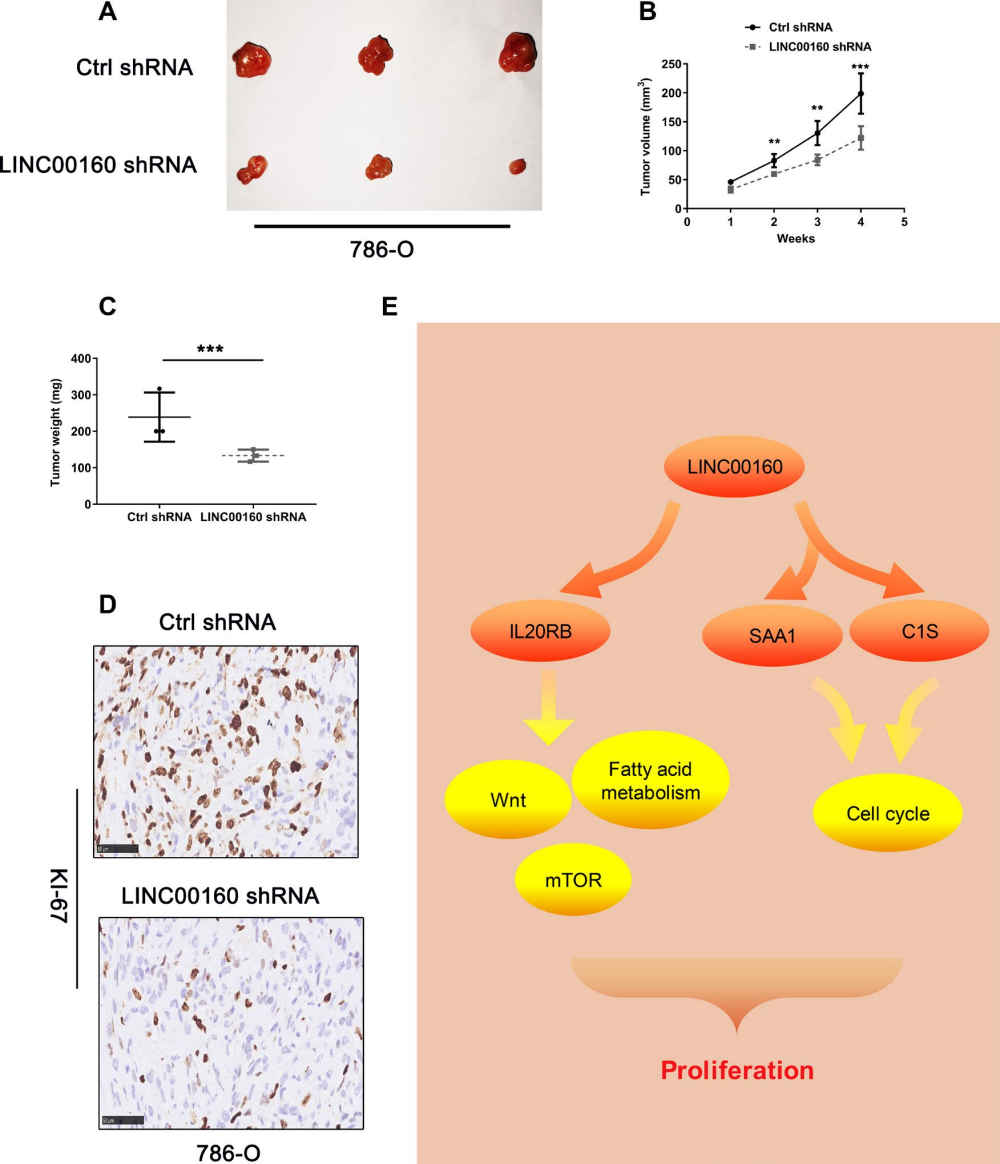
**The knockdown of LINC00160 levels inhibits tumorigenesis in *vivo*.** (**A**–**C**) Tumor size, volume curves, and weight in the xenograft formation assay, (**D**) The expression of Ki67 protein in xenograft tumor tissues. (**E**) Schematic diagram of LINC00160 in ccRCC. Data was represented as the mean±SEM. *P <0.05, **P<0.01, ***P<0.001 and ****P<0.0001.

We examined the effect of knockdown LINC00160 on renal tumor growth in *vivo*. 786-O cells stably infected with lentivirus shRNA-LINC00160 and implanted into flank of nude mice. We uncovered that LINC00160 knockdown in 786-O cells significantly decreased tumor weight and tumor volume (Figure 7A–7C). IHC staining showed that knockdown LINC00160 reduced Ki67 protein expression (Figure 7D). Collectively, these results indicated that LINC00160 silencing could inhibit renal cancer growth.

## DISCUSSION

Clear cell renal cell carcinoma (ccRCC) accounts for approximately 80% of all RCC histological types and own high tendency towards invasion, metastasis, and resistant to chemotherapy [24, 25]. It is now axiomatic that lncRNAs exert crucial effect on tumor occurrence, progression, metastasis, and drug resistance [26]. Nevertheless, functions of abundant lncRNAs’ participation in tumor are largely unknown. Our study reported the role of LINC00160 expression in clinical significance and biological functions of ccRCC for the first time.

Genomic location for LINC00160 gene is chromosome 21; in breast cancer, Jonsson P et al. showed that silencing of LINC00160 resulted in reduced proliferation [27]. In the present study, public database and our experimental evidence revealed that LINC00160 was significantly upregulated in ccRCC, and that high expression of LINC00160 predicted poor outcome and was correlated with high T stage, grades, and TNM stage in TCGA_KIRC cohort; moreover, knockdown of lncRNAs in ACHN and 786-O cells impaired cell proliferation, migration, and invasion, whereas overexpression of lncRNAs accelerated cell proliferation, migration, and invasion in vitro; the xenograft tumor model in vivo indicated that the knockdown of LINC00160 level inhibits tumorigenesis. These results together demonstrated that LINC00160 acted as an oncogenic factor in ccRCC and promoted tumor progression.

lncRNAs in urologic cancers functions as versatile regulators of major cellular pathway controlling proliferation, apoptosis, and pathogenesis of human malignancies [11]. Previous research in lncRNAs partially unravel this enigma that lncRNAs could regulate gene expression epigenetically as Cis or Trans-acting repressor and transcriptional activator, such as PCAT1, HOTAIR, and PRNCR1 [28–30]; moreover, lncRNAs could function as microRNA sponge, protein-interacting competing endogenous RNAs, and functional pseudogenes [31, 32]. In our study, the separation of nuclear and cytoplasmic RNA assays revealed that LINC00160 was predominantly localized in nucleus in two cell lines, which indicates that LINC00160 probably perform transcriptional regulation role to exert its influence on tumor progression. LINC00160 was shown to be direct transcriptional targets of estrogen receptor-α in breast cancer [27]. However, we did not elucidate the specific regulation mechanism with regard to overexpression of LINC00160 in ccRCC.

To investigate its possible underlying mechanism, we conducted the correlation analysis between LINC00160 expression and protein-coding genes. C1S, CDA, SAA1, PANX2, IL20RB, and TPSG1 showed the correlation coefficient were up to 0.5. GSEA demonstrated that high C1S and SAA1 expression were significantly associated with cell cycle pathway in patients with ccRCC. It may be hypothesized that LINC00160 could positively promote cell cycle progression in ccRCC via C1S or SAA1. Despite our experiments in vitro and vivo, further experiments need to be verified. In addition, GSEA also indicated that low IL20RB expression was significantly associated with Wnt, mTOR signaling pathway, fatty acid metabolism in patients with ccRCC. The fatty acid metabolism in ccRCC attract much attention and was confirmed to promote tumor progression [33, 34]. We hypothesized that LINC00160 could positively promote metastasis in ccRCC via IL20RB. Further experiments are still needed. One limitation in our study is that LINC00160 is not only upregulated in ccRCC samples but also overexpressed in sunitinib resistant cells, we only explored its roles in ccRCC, but we did not investigate its drug resistant roles in this study. In the future, we will investigate its roles in drug resistance.

This study is the first study to demonstrate the functional role of LINC00160 in ccRCC tumor progression. These results also reveal that LINC00160 could be treated as a novel biomarker predicting the prognosis of ccRCC patients. High expression of LINC00160 is significantly positively associated with ccRCC progression. All of these studies provided clues to a new therapeutic target for the management of ccRCC patients.

## MATERIALS AND METHODS

### Cell culture

The human renal proximal tubular epithelial cell line (HK-2) and the 786-O, OS-RC-2, ACHN and Caki-1 human RCC cell lines were purchased from the American Type Culture Collection (Manassas, VA, USA). Cells were maintained at 37°C in a 5% CO2 incubator and cultured in high glucose Dulbecco's modified Eagle's medium (Thermo Fisher Scientific, Inc., Waltham, MA, USA), containing 10% fetal bovine serum (Thermo Fisher Scientific, Inc.) and 1% penicillin-streptomycin.

### Clinical sample preparation

Between 2016 and 2018 at the Department of Urology, Union Hospital (Wuhan, China), eighteen paired kidney cancer and adjacent normal tissues were collected from patients who underwent radical nephrectomy (Supplementary Table 1). The patients whose tissues were used in the present study had never received chemotherapy or radiotherapy. The specimens were stored at -80°C until use. The study protocol was approved by the ethics committee of the Union Hospital and Huazhong University of Science and Technology, and all patients provided written informed consent. The study methodologies conformed to the standards set by the Declaration of Helsinki.

### Separation of nuclear and cytoplasmic RNA assay

Separation of nuclear and cytoplasmic RNA was performed using PARIS kit (Thermo Fisher Scientific, Inc.) according to the manufacturer’s instruction. GAPDH and U6 were used as cytoplasmic and nuclear control separately.

### qRT-PCR

Total RNA was extracted using TRIzol reagent (Thermo Fisher Scientific, Inc.). RNAs were reverse-transcribed to cDNA using a PrimeScript RT reagent Kit (Takara Biotechnology Co., Ltd., Dalian, China). qRT-PCR was performed using the ABI StepOnePlus system (Thermo Fisher Scientific, Inc.). Relative expression was calculated using the -2ΔΔCt method, and GAPDH was used as the internal control. The PCR primers used were as follows: GAPDH, AAAAGCATCACCCGGAGGAGAA forward and AAGGAAATGAATGGGCAGCCG reverse; LINC00160, ACAGCCAACCACCCATTCTCTT forward and AGGGAAGGCAGCAGACAAAACC reverse; U6 primers were synthesized by Ribobio (Guangzhou, China).

### Cell infection and transfection

Expression lentivirus for LINC00160 short hairpin RNA (LINC00160 shRNA) (Vigene Biosciences, USA) and negative control (Vigene Biosciences, USA) were transfected in ACHN and 786-O cell lines at a multiplicity of infection (MOI) of 15 assisted with ADV-HR (Vigene Biosciences, USA) according to the manufacturer’s instructions. For plasmids transfections, cells were cultured in six well plants and transfected with 4 μg expression plasmids for LINC00160 shRNA (Vigene Biosciences, USA), Ctrl shRNA (Vigene Biosciences, USA), LINC00160 (Genechem, China), Vector (Genechem, China) using Lipofectamine 2000 (Thermo Fisher Scientific, Inc.) according to the manufacturer’s instructions.

### Cell counting kit-8 (CCK-8) assay

Cells were seeded into 96-well plates at a density of 1000 cells per well. CCK-8 reagents (Djingo, Japan) were added into wells at 24, 48, 72, 96 hours. The optical density was measured at a wavelength of 450 nm.

### Transwell assay

Cell invasion assays were performed using 8 μm pore-size chambers coated with 60 μl matrigel gel (Corning, Inc., NY, USA). 2 × 104 cells were resuspended in serum-free medium and seeded into the upper chambers; the bottom chambers were filled with medium containing serum. After incubation for 24 hours, the invasive cells were stained and imaged under 100X magnification. Five random fields were analyzed for each chamber. Cell migration assays were performed using chambers without matrigel gel. 1.5 × 10^4^ cells were resuspended in serum-free medium and other procedures were the same as above.

### Wound healing assay

When confluence reached 90% in 6-well plates, a 10 μl pipette tip was used to scratch on the monolayer. Then, cells were washed and starved to migrate for indicated time. Images were taken at 0, 24, 36 hours under 40X magnification.

### Differentially expressed gene exploration

GSE profiles from GEO DataSets (https://www.ncbi.nlm.nih.gov/gds/) were used for screening. The Student's t-test was used to analyze differences in the lncRNA expression between 54 paired samples. The lncRNA expression pattern and clinicopathological characteristics were downloaded from the TANRIC (https://ibl.mdanderson.org/tanric/_design/basic/main.html) and The Cancer Genome Atlas Kidney Clear Cell Carcinoma (TCGA-KIRC; https://xenabrowser.net/heatmap/) databases, respectively. Kaplan-Meier curves with log-rank tests were used to assess the correlation between the lncRNA expression and disease-free survival (DFS) and overall survival (OS) rates. Samples from the TCGA-KIRC database were divided into two groups, according to the median expression of candidate lncRNA. Receiver operating characteristic curve (ROC) and areas under the curve (AUC) were generated to evaluate the diagnostic values of lncRNAs in ccRCC. The association between lncRNA expression and clinicopathological parameters in ccRCC was evaluated using Pearson’s test. Annotations for candidate lncRNAs were retrieved from GeneCards (https://www.genecards.org/) and Ensembl genome browser 90 (http://asia.ensembl.org/index.html).

### Bioinformatics analysis

Protein-coding potential was assessed on LNCipedia (https://lncipedia.org/) and lncRNAtor (http://lncrnator.ewha.ac.kr). Location of genes were predicted on lncATLAS (http://lncatlas.crg.eu/) and GeneCards (https://www.genecards.org/). GSEA was performed to understand the pathway involved in the pathogenesis of TCGA_KIRC. Nominal *P* < 0.05 and a false discovery rate < 25% were considered as significantly enriched gene set analysis.

### Xenograft tumor in nude mice and immunohistochemistry (IHC)

All animal experiments were conducted in accordance with animal protocols approved by the Institutional Animal Use and Care Committee of the affiliated Union Hospital of Huazhong University of Science and Technology. Six male nude mice were randomly divided into two groups and a total of 1 × 10^6^ 786-O cells with sh-LINC00160 or NC were injected into the flank of mice subcutaneously. Tumor size was measured every 3 days. Tumor volumes were calculated by (D × d^2^)/2. The performer was blinded to the group allocation during the experiment. The mice were euthanized and the tumors were harvested after 30 days. Then, six xenograft tumor were fixed in formalin, dehydrated, and embedded. The tissue sections were incubated with primary rabbit Ki67 (1:100; A11005; ABclonal Biotech Co., Ltd) and then incubated with secondary antibody for 2h at room temperature.

### Statistics

Statistical analysis was carried out using Graphpad Prism 7.0 software, and each experiment was performed at least three times. The Student's t-test was used to analyze differences in the gene expression between two groups. Data is presented as the mean ± SEM, and *P*<0.05 was considered to indicate a statistically significant difference.

### Availability of data and materials

The datasets used and/or analyzed during the current study are available from the corresponding author on reasonable request.

### Ethics approval and consent to participate

The present study and experimental procedures were approved by the Human Research Ethics Committee of Huazhong University of Science and Technology (Wuhan, China). Written informed consent was obtained from the patients/patients' families. The study was conducted according to the principles outlined in the Declaration of Helsinki.

## Supplementary Material

Supplementary Figures

Supplementary Table 1
