# Supplementary material for: Overexpression of LINC00160 predicts poor outcome and promotes progression of clear cell renal cell carcinoma
Source: Aging (Albany NY). 2020 Apr 21;12(8):7448–64. doi: 10.18632/aging.103091 (PMC7202521; doi:10.18632/aging.103091)
Supplement: Supplementary Figures [file aging-12-103091-s002..pdf]

SUPPLEMENTARY FIGURES

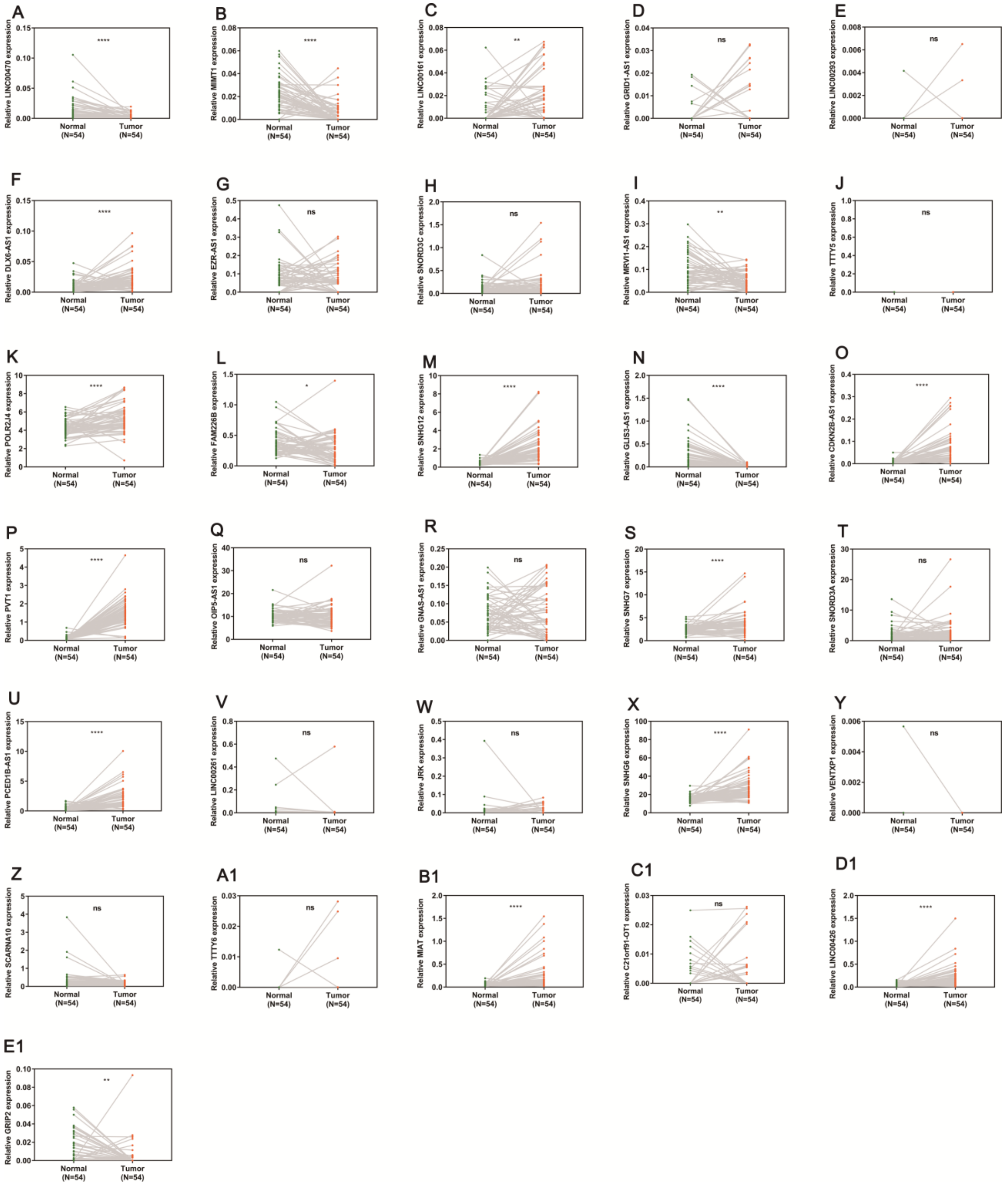

Supplementary Figure 1. Paired student's *t*-tests of the 36 upregulated lncRNAs. (**A**–**E1**) Relative gene expression comparison of 54 paired tissues in the TCGA-KIRC database.

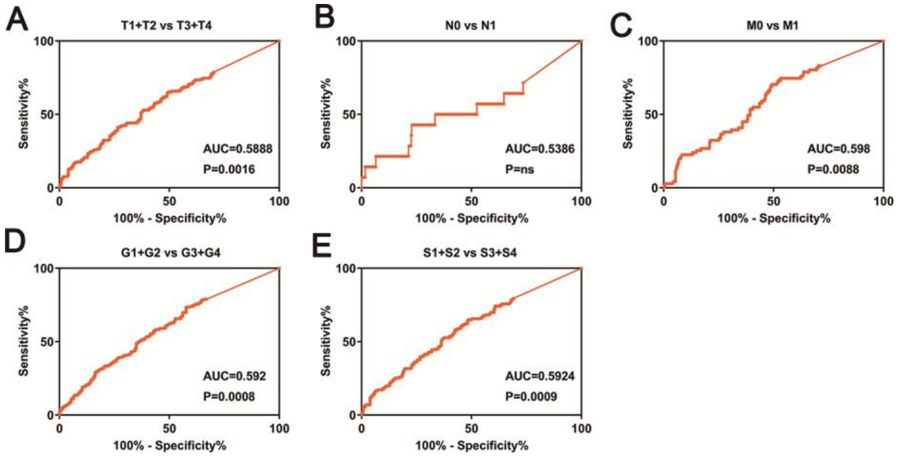

Supplementary Figure 2. ROC curves were used to distinguish between the G1+G2 and G3+G4, M0 and M1, N0 and N1, T1+T2 and T3+T4, and Stage I+II and Stage III+IV patient samples. (**A**) T1+T2 versus T3+T4. (**B**) N0 versus N1. (**C**) M0 versus M1. (**D**) G1+G2 versus G3+G4. (**E**) Stage I+II versus Stage III+IV.
