# Supplementary material for: Overexpression of LINC00160 predicts poor outcome and promotes progression of clear cell renal cell carcinoma
Source: Aging (Albany NY). 2020 Apr 21;12(8):7448–64. doi: 10.18632/aging.103091 (PMC7202521; doi:10.18632/aging.103091)
Supplement: Supplementary Table 1 [file aging-12-103091-s001..pdf]

SUPPLEMENTARY TABLE

Supplementary Table 1. Basic characteristics of 18 patients with ccRCC.

Characteristic	N (%)
Age	
Mean ± SEM, y	56±11
Gender	
Male/female	12/6
Tumor size	
Mean ± SEM, cm	9.2±3.4
Location	
Right/left	10/8
T stage	
T1b	4(22.22)
T2a	6(33.33)
T2b	5 (27.77)
T3	2 (11.11)
T4	1 (5.55)
N stage	
N0	16 (88.89)
N1	2(11.11)
M stage	
M0	18 (1)
M1	0 (0)
Fuhrman grade	
1	4(22.22)
2	8(44.44)
3	4 (22.22)
4	2(11.11)
Chronic Kidney Disease (CKD)	0
